# Functional Enrichment Analysis of Rare Mutations in Patients with Brain Arteriovenous Malformations

**DOI:** 10.3390/biomedicines13061451

**Published:** 2025-06-12

**Authors:** Elena Zholdybayeva, Ayazhan Bekbayeva, Karashash Menlibayeva, Alua Gusmaulemova, Botakoz Kurentay, Bekbolat Tynysbekov, Almas Auganov, Ilyas Akhmetollayev, Chingiz Nurimanov

**Affiliations:** 1 National Center for Biotechnology, Astana 010000, Kazakhstan; info@biocenter.kz (A.B.); alua.gusmaulemova6@gmail.com (A.G.); bkurentay@mail.ru (B.K.); tynysbekovw1@gmail.com (B.T.); auganov.almas@gmail.com (A.A.); iliyas@mail.ru (I.A.); 2 Department of Vascular and Functional Neurosurgery, National Centre for Neurosurgery, Astana 010000, Kazakhstan; karashash.2.menlibayeva@kcl.ac.uk (K.M.); chingiz198705@gmail.com (C.N.); 3 Department of Population Health Sciences, Faculty of Life Sciences and Medicine, King’s College London, London SE1 1UL, UK

**Keywords:** brain arteriovenous malformation, exome sequencing, mutation, candidate genes, enrichment analysis

## Abstract

**Background/Objectives:** Brain arteriovenous malformations (bAVMs) are rare vascular anomalies characterized by direct connections between arteries and veins, bypassing the capillary network. This study aimed to identify potential genetic factors contributing to the development of sporadic bAVMs. **Methods**: Three patients (AVM1–3) from Kazakhstan who underwent microsurgical resection at the National Centre for Neurosurgery (NCN) in Astana, Kazakhstan, were analyzed. Brain AVMs were diagnosed using magnetic resonance imaging (MRI). Genomic DNA was isolated from whole venous blood samples, and whole-exome sequencing was performed on the NovaSeq 6000 platform (Illumina). Variants were filtered according to standard bioinformatics protocols, and candidate gene prioritization was conducted using the ToppGene tool. **Results**: In silico analysis further revealed candidate genes likely associated with lesion development, including COL3A1, CTNNB1, LAMA1, NPHP3, SLIT2, SLIT3, SMO, MAPK3, LRRK2, TTN, ERBB2, PARD3, and OBSL1. It is essential to focus on the genetic variants affecting the following prioritized genes: ERBB2, SLIT3, SMO, MAPK3, and TTN. Mutations in these genes were predicted to be “damaging”. Most of these genes are involved in signaling pathways that control vasculogenesis and angiogenesis. **Conclusions**: Defects in genes associated with ciliary structure and function may be critical to the pathogenesis of brain AVMs. These findings provide valuable insights into the molecular underpinnings of bAVM development, emphasizing key biological pathways and potential candidate genes. Further research is needed to establish robust correlations between specific genetic mutations and clinical phenotypes, which could ultimately inform the development of improved diagnostic, therapeutic, and prognostic approaches.

## 1. Introduction

Brain arteriovenous malformations (bAVMs) are rare vascular anomalies characterized by direct connections between arteries and veins, bypassing the capillary network [1]. These malformations occur in approximately 1 in 100,000 people annually and present a significant risk of rupture, which can result in potentially fatal cerebral hemorrhage, loss of consciousness, and severe neurological deficits [2]. The mortality rate associated with bAVM rupture ranges from 10% to 15% [3], while the risk of hemorrhage-induced disability varies between 10% and 40% [4].

The primary objective of bAVM treatment is to prevent rupture and reduce the risk of disability or death.

Arteriovenous malformations (AVMs) were previously considered congenital defects of the cerebral vasculature. However, with the adoption of genomic and other multi-omics technologies over the past few decades, perspectives on brain malformations have evolved significantly. Numerous findings have emerged indicating the possibility of AVM development during the post-embryonic period, as well as evidence of substantial changes in malformation characteristics over time. Currently, AVMs are classified into hereditary and sporadic forms. More than 97% of cases are sporadic, while approximately 3% of AVMs are associated with hereditary hemorrhagic telangiectasia (HHT), an autosomal dominant disorder also known as Rendu–Osler–Weber disease [5]. HHT is caused by mutations in the ENG (OMIM: 131195), ACVRL1 (OMIM: 601284), and SMAD4 (OMIM: 600993) genes. All three genes encode proteins involved in the transforming growth factor beta (TGF-β) signaling pathway [6].

Studies suggest that additional genes responsible for HHT exist but have yet to be identified; these genes are thought to be located on chromosomes 5 and 7 [7]. The hereditary form of AVM also includes capillary malformation–arteriovenous malformation (CM-AVM, OMIM #608354), which is caused by mutations in the RASA1 gene [8]. The development of sporadic bAVMs is generally attributed to a combination of environmental and genetic factors. Growing evidence supports a genetic contribution to the occurrence of sporadic bAVMs [9]. Many AVMs are sporadic, and SNPs in some specific genes are responsible for the sporadic susceptibility to bAVMs [10].

However, the pathogenesis of nonhereditary bAVMs remains poorly understood [11]. A more comprehensive understanding of the molecular mechanisms underlying these malformations is essential for the development of targeted therapies and the early identification of high-risk patients. Genetic studies have elucidated possible mechanisms contributing to the development of bAVMs, with emerging evidence indicating that mutations in critical signaling pathways play a pivotal role in their pathogenesis [12].

Whole-exome sequencing (WES), a next-generation sequencing (NGS) technology, has significantly advanced the understanding of genetic factors involved in bAVMs by enabling the identification of mutations in protein-coding regions of the genome. Several studies have identified rare genetic variants linked to bAVM pathogenesis, including mutations within the BMP/TGF-β and VEGF/VEGFR signaling pathways [13,14,15,16]. Nonetheless, the genetic landscape of bAVMs remains heterogeneous, and further research is needed to elucidate the specific germline mutations that contribute to their formation.

The present study is the first pilot study in Central Asia to investigate sporadic bAVMs using whole-exome sequencing. It aims to investigate rare germline mutations associated with the development of bAVMs by leveraging WES technology to identify novel genetic variants that may contribute to their pathogenesis. By identifying these mutations, this research seeks to advance our understanding of bAVM biology and support the development of more effective diagnostic and therapeutic strategies.

## 2. Materials and Methods

### 2.1. Patient Recruitment

**Figure 1:**
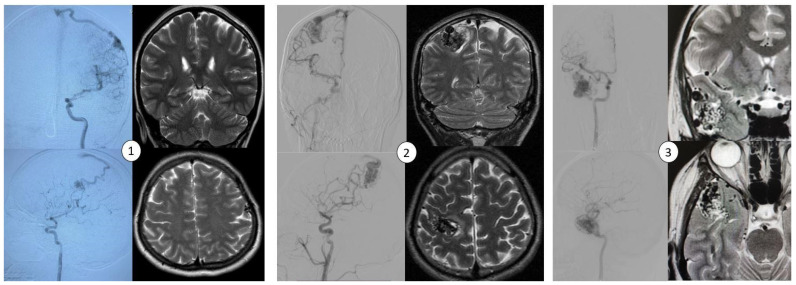
Neuroradiological imaging findings of bAVM lesions, including cerebral angiograms in both anterior and lateral projections and MRI scans (T2-weighted sequences) in the axial and coronal planes. The images correspond to the three patients described in the study: (1) AVM1—located in the left frontal lobe, (2) AVM2—located in the right parietal lobe, and (3) AVM3—located in the right temporal lobe.

This study was conducted in a cohort of three patients (AVM1–3) who underwent microsurgical resection of bAVMs at the National Centre for Neurosurgery (NCN) in Astana, Kazakhstan. bAVMs were diagnosed using magnetic resonance imaging (MRI) and digital subtraction angiography (DSA) performed using a biplane system (Artis Zee Biplane System, Siemens, Erlangen, Germany) (Figure 1). Diagnostic imaging was reviewed by a multidisciplinary team of neuroradiologists and neurosurgeons. The diagnoses were confirmed through histopathological analysis of resected specimens.

A Spetzler–Martin grading system was used to assess lesion severity, assigning a score based on the angiographic features of the bAVMs to predict the morbidity and mortality risk associated with surgery. None of the patients reported a family history of bAVM, so they were classified as sporadic cases. The exclusion criterion was a known diagnosis of hereditary hemorrhagic telangiectasia, capillary malformation–arteriovenous malformation (CM-AVM), Sturge–Weber syndrome, or another Mendelian vascular disorder.

All participants were fully informed about their inclusion in the study, and informed consent was obtained from adult participants and legal guardians of underage patients. This manuscript does not contain identifying information. This study was approved by the ethics committee of the National Center for Biotechnology (#9/07.011.2022, Nur-Sultan, Kazakhstan) and was conducted according to the principles expressed in the Declaration of Helsinki.

### 2.2. DNA Isolation and Whole-Exome Sequencing

Genomic DNA was isolated from 9 mL of EDTA-anticoagulated whole venous blood using a standard salt-out method [17]. Quantitative analysis of DNA concentrations was initially performed using a NanoDrop 1000 spectrophotometer (Thermo Fisher Scientific, Waltham, MA, USA). For more precise measurements, an Qubit 2.0 Fluorometer (Invitrogen; Life Technologies, Carlsbad, CA, USA) was used to determine DNA concentrations, as these nucleic acids were intended for WES.

To generate standard exome capture libraries, a SureSelect V6-Post kit (Agilent Technologies, Santa Clara, CA, USA) for an Illumina paired-end sequencing library was used with 1 µg of input gDNA. Whole-exome sequencing was performed using the Novaseq 6000 platform (Illumina Inc., San Diego, CA, USA), following the manufacturer’s instructions.

### 2.3. Bioinformatic and Statistical Analyses

The base calling files, which were expressed in binary, were converted into FASTQ using Illumina package bcl2fastq v2.20.0. Paired-end sequences produced via the NovaSeq Instrument were first mapped to the human reference genome using the mapping program BWA Version bwa-0.7.17 (https://sourceforge.net/projects/bio-bwa/, accessed on 24 December 2024). The Mapping Reference hg38 from UCSC (original GRCh38 from NCBI, December 2013) was used. Duplicate reads were removed with Picard–tools—Version 2.18.2-SNAPSHOT. Genetic variants were identified using the Genome Analysis Toolkit (GATKv4.0.5.1) (https://gatk.broadinstitute.org/hc/en-us, accessed on 25 December 2024), a robust software framework designed for high-throughput sequencing data analysis. Filtered variants were annotated with another program called SnpEff (SnpEff 4.3t 2017-11-24) (https://sourceforge.net/projects/snpeff/files/snpEff_v4_3s_core.zip/download, accessed on 25 December 2024) and filtered with dbSNP and SNPs from the 1000 Genomes project. The final product was in vcf format. The in-house program and SnpEff were then used for annotation with additional databases, including ESP6500, ClinVar, dbNSFP, and ACMG information.

### 2.4. Variant Filtering Criteria

The variants were filtered based on several criteria. Variants showing a Phred Quality Score < 40 were discarded (in Excel). In this study, the probability of an incorrect base call was 1 in 10,000 (a base call accuracy of 99.9%). Using this threshold, subsequent validation via Sanger sequencing confirmed the findings. When filtering, only rows with ‘PASS’ in the ‘FILTER’ column and only ‘protein_coding’ in the ‘Transcript_BioType’ column were left. Filtered variants were classified by functional class, and intronic, synonymous, non-coding RNA, and untranslated regions affecting variants were discarded.

Missense, nonsense, frameshift, and short indels presenting a minor allele frequency (MAF) < 0.01 were selected. For MAF-based filtering, we used the values reported in the Genome Aggregation Database (https://gnomad.broadinstitute.org/, accessed on 25 December 2024) [18] and those reported in phase 3 of the 1000 Genomes project [19].

### 2.5. Gene Ontology Analysis and Prioritization of Genes

The data were functionally annotated using bioinformatic tools. To visualize and functionally group genes containing filtered genetic variants, we used the ClueGO plugin (version 2.5.10). Cytoscape (version 3.8.0) software was used for each sample [20]. Clustering was performed based on the GO Biological Process, KEGG Pathways, REACTOME, and WikiPathways ontologies. Groups showing Bonferroni step-down corrected *p*-values ≤ 0.05 were considered significant. Next, ToppGene was used for candidate gene prioritization (https://toppgene.cchmc.org/, accessed on 4 February 2025); this website is free, open to all users, and does not require a login to access). The ToppGene algorithm has been described by Chen and colleagues [21]. Genes within the chosen groups were added to the Test Gene Set in ToppGene. Candidate genes were ranked based on functional similarity to the list of training genes. The training gene set group consisted of genes that have been confirmed to cause HHT and a few familiar bAVM cases without HHT (genes: ENG, ACVRL1, TGFBR2, SMAD4, and dGDF2). The training parameters selected were “GO:Biological Process”, “Human Phenotype”, “MousePhenotype”, “Pathway”, “PubMed”, “Interaction”, and “Disease”. Statistical parameters were calculated using the Bonferroni correction, and *p*-values ≤ 0.05 were considered significant.

### 2.6. Sanger Validation

Variants carried by prioritized genes were validated via Sanger sequencing using the BigDyeTerminator© v3.1 Cycle Sequencing Kit (Applied Biosystems, Vilnius, Latvia) and a 3730 XL Genetic Analyzer (Applied Biosystems, Waltham, MA, USA). Polymerase chain reaction (PCR) primers were designed; the PCR conditions are provided in Appendix A.1, Table A1 and Table A2).

## 3. Results

### 3.1. Patient Recruitment Data

This study was performed in a group of three Kazakh patients diagnosed with bAVM. Anamnestic data for the patients are provided in Table 1.

### 3.2. WES Results Analysis

Basic summary statistics of the raw sequence data obtained from the study samples is presented in Table 2 (the fastq files relating to the samples can be provided upon request).

The detailed alignment metrics for each sample, the depth of the coverage summary, and variant metrics for all samples are provided in Table 3.

After applying the above filtering criteria, genetic variants in the genes for each sample were selected. Ultimately, there were 314 genetic variants for AVM1, 321 for AVM2, and 312 for AVM3. Full lists are available in the Supplementary Materials (Table S1—AVM1; Table S2—AVM2; Table S3—AVM3).

### 3.3. Results of Gene Ontology Analysis and Prioritization of Genes

Using ClueGO (Gene Ontology) version 2.5.10 software, an enrichment analysis tool, genes with potentially pathogenic variants were grouped for each exome to identify the biological pathways and processes they are involved in. A significant functional enrichment was observed. The results are summarized in Table 4, with a Bonferroni-adjusted *p*-value of ≤0.05.

As can be seen from Table 4, the studied samples presented ontologies related to the formation and mobility of microtubules (GO:0036159, GO:0070286), cell morphogenesis (GO:0048667), cell migration (GO:1904417), extracellular transport (GO:0006858, GO:0003351), the development of the cardiovascular system (GO:0055013), and signaling pathway regulation (GO:2001044). Biological processes not related to general cellular functions, signaling pathways, or intra-/extracellular transport were excluded from further analysis. Gene prioritization is the process of assigning similarity or confidence scores to genes and ranking them based on the probability of their association with the disease of interest.

The next step involved gene prioritization using ToppGene to identify the candidate genes that are most likely to be functionally significant in the development of bAVM. For gene prioritization, which is an effective and commonly used approach for identifying potential gene–disease associations, this study used the algorithm presented by Concetta Scimone et al. (2020) [22]. The algorithm is described in the Materials and Methods section. The purpose of gene prioritization was to link the selected loci with others that are already associated with the development of bAVM. For each sample, the set of training genes consisted of the clustered genes obtained, shown in Table 4 (seventh column). In Appendix A.3, only genes with an overall *p*-value ≤ 0.05 are presented for each sample (AVM1, AVM2, and AVM3). These are DNAAF2, LAMA1, NPHP3, DNAAF1, and CCDC40 for AVM1, and PRODH for AVM2, CTNNB1, COL3A1, ERBB2, SMO, MAPK3, TTN, PARD3, RNF31, CTBP2, SLIT2, CDH23, NRP2, ARHGEF25, CELSR2, LRRK2, SLIT3, and OBSL1.

Thus, only genes showing an overall *p*-value ≤ 0.05 (Appendix A.3) with the criterion “Gene Ontology (GO) biological process” annotations were prioritized for all studied exomes (Table 5).

The genetic variants affecting the prioritized genes are presented in Table 6. All these variants were confirmed via Sanger sequencing. Primer sequences selected for the genetic variants (from Table 6) are presented in the Table A1 in Appendix A.1.

It is essential to focus on the genetic variants affecting the prioritized genes ERBB2, SLIT3, SMO, MAPK3 and TTN. Mutations in these genes were predicted to be “damaging” according to the SIFT_pred algorithm (Table 6).

### 3.4. Results of Sanger Validation

**Figure 2:**
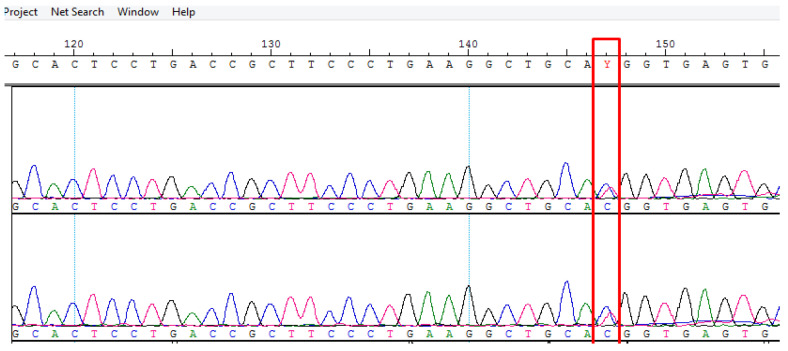
Validation analysis of the candidate variant in the gene SMO (c.536C>T; rs115491500).

**Figure A1:**
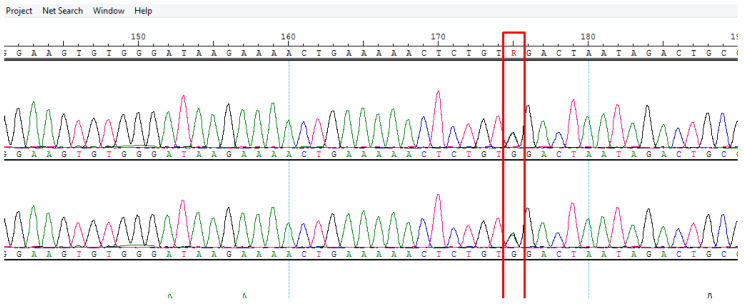
Validation analysis of the candidate variants (p.Gly2385Arg, rs34778348) in the genes LRRK2.

The candidate genetic variant was further validated through Sanger sequencing. Figure 2 provides an example of an electropherogram for the prioritized gene; additional electropherograms are provided in the Appendix A.2 (Figure A1). This variant was validated in 10 healthy individuals to eliminate any false-positive findings. MRI was performed for these individuals to exclude bAVM.

## 4. Discussion

Sporadic forms of bAVMs account for approximately 95–97% of all cases, highlighting the complexity and multifactorial nature of their pathogenesis [23,24]. Genotyping of cohorts with sporadic bAVMs has yet to consistently identify single-nucleotide polymorphisms or chromosomal structural variations that contribute to AVM development, leaving the precise mechanisms of pathogenesis unclear. However, certain key factors have been established, including the involvement of angiogenic factors and inflammatory cytokines in the development of bAVMs [9].

Recent advancements in high-throughput sequencing techniques have provided deeper insights into the genetic underpinnings of bAVMs. A 2021 review by Hans-Jakob Steiger summarized key studies on bAVM pathogenesis, highlighting significant progress in understanding the genetic factors and pathophysiology of cerebral AVMs [25]. Whole-genome sequencing has substantially advanced our knowledge of the genetic origins of sporadic and familial AVMs; however, several aspects of their pathogenesis remain unresolved. A 2023 review by Krisna Maddy summarizes the genetic causes of bAVM development. Genetic variants identified in patients with bAVMs are involved in the transforming growth factor beta-1 (TGF-β1) signaling pathway, angiogenesis regulation, and arterial and venous differentiation. Mutations in the KRAS and BRAF genes in bAVM patients are associated with endothelial proliferation, angiogenic signaling, or vascular remodeling processes. The most common mutation associated with AVMs is MAP2K1-K57N. It has been suggested that mutant endothelial cells with MAP2K1 mutations interfere with normal vascular development and may lead to abnormal arteriovenous connections [10].

The role of non-coding RNAs in the pathogenesis of AVMs is also receiving increased attention. For example, three critical microRNAs (miRNAs) involved in VEGF signaling were identified in the blood samples of patients with bAVM compared to healthy individuals.

Epigenetic mechanisms are also involved in the development of bAVMs.

Whole-exome sequencing was performed in a Chinese population. WES was performed on the patients and their phenotypically normal parents [16]. An increasing number of studies based on data from a single whole-exome sequencing analysis are being published, such as the study by Scimone and colleagues [14]. WES analysis was conducted in a child of European descent diagnosed with sporadic bAVM. In a study by Kun Wang, whole-exome sequencing of blood-derived DNA was performed in a cohort of 150 patients of Chinese origin with bAVM [26]. The identification of novel mutations and genes through high-throughput sequencing has improved our understanding of the pathogenic mechanisms underlying brain arteriovenous malformations (bAVMs). Huan Huang et al. were the first to report the potential involvement of heterozygous mutations in the NOTCH2 gene in the pathogenesis of AVMs. A case was described in which a child diagnosed with bAVM at age 7 experienced a hemorrhage at age 12, followed by another episode one year later due to disease progression. The NOTCH2 protein functions as a receptor in the Notch signaling pathway, which is important in regulating angiogenesis [27].

The replication of previously identified associations in new populations, along with the investigation of genes linked to related phenotypes, represents a key approach to uncovering the genetic contribution to complex multifactorial diseases. In our study, we conducted whole-exome sequencing on three patients aged between 25 and 40 years, all of whom were diagnosed with sporadic bAVM, employing a research approach similar to that of Concetta Scimone et al. [9,22]. Through in silico analyses, we identified key biological pathways enriched in germline-mutated genes. While the specific mutated genes varied across patients, the biological pathways involved demonstrated significant consistency. These findings align with those reported by Scimone et al. [14]. Specifically, enrichment analysis revealed pathways related to integrin-mediated signaling, epithelial cilium movement associated with extracellular fluid, and extracellular transport in AVM1; glutamate metabolism and the regulation of CD4 production in AVM2; and, more broadly, pathways involved in the positive regulation of intracellular signaling, cell migration, cell differentiation, vasculature development, and tube formation.

We performed priority gene analysis for each sample to identify the genes most strongly associated with the development of bAVMs.

### 4.1. AVM1

For the AVM1 sample, significant loci (*p* < 0.05) associated with phenotypes and pathways identified using the ToppGene tool included DNAAF2, LAMA1, NPHP3, DNAAF1, and CCDC40. DNAAF2 encodes a highly conserved protein essential for the cytoplasmic preassembly of axonemal dyneins, playing a crucial role in the motility of cilia and flagella. DNAAF1 encodes a cilia-specific protein required for the structural stability of cilia. It participates in the cytoplasmic preassembly of dynein arms and regulates both microtubule-based cilia and actin-based microvilli. LAMA1 encodes a protein that interacts with cells via the high-affinity receptor laminin, mediating cell adhesion, migration, and tissue organization during embryonic development through interactions with extracellular matrix components. NPHP3 encodes a protein necessary for the proper development and function of cilia. CCDC40 encodes a protein central to the motility of cilia and flagella. Collectively, these genes are involved in biological processes such as regionalization, tissue morphogenesis, heart morphogenesis, blood vessel morphogenesis, and tube morphogenesis. Cheong et al. (2019) demonstrated the expression of DNAAF1 in mouse embryos during various developmental stages and multiple adult mouse tissues, including the brain [28].

Enrichment analysis of the AVM1 sample highlighted significant ontologies related to ciliary and tubular organization. Previous studies have documented the extensive expression of cilia in endothelial cells during early vasculogenesis. Shahram Eisa-Beygi et al. examined the role of cilia in regulating early cranial vessel morphogenesis, proposing a critical, flow-independent function of endothelial cell (EC) cilia in the brain, which is vital for cerebrovascular stability. Dysfunction of ciliary processes may thus contribute to the pathogenesis of bAVMs [29].

The vascular barrier separating blood from tissues is highly selective and essential for maintaining tissue homeostasis. Defects in this barrier contribute to various cardiovascular diseases, emphasizing the critical role endothelial cells play in maintaining barrier integrity [30]. Our findings align with those reported by Concetta Scimone et al., suggesting that defects in genes responsible for ciliary assembly may be involved in bAVM development [14].

### 4.2. AVM2

For this sample, the following priority genes were identified: PRODH, ACOT8, and FTCD.

The PRODH gene encodes a mitochondrial protein that catalyzes the initial step in proline degradation. The protein encoded by the ACOT8 gene is a peroxisomal thioesterase that is primarily involved in fatty acid oxidation rather than synthesis. The FTCD gene encodes a bifunctional enzyme responsible for transferring one-carbon units from formiminoglutamate, a metabolite in the histidine degradation pathway, into the folate pool.

Regarding the potential role of the PRODH gene in vascular formation or bAVM pathogenesis, the gene prioritization analysis conducted via ToppGene only references one publication [31] that presents a high-resolution spatiotemporal atlas of gene expression in the developing mouse brain. However, there is no information currently available concerning the involvement of the ACOT8 and FTCD genes in vascular development or bAVM pathogenesis.

### 4.3. AVM3

The highest number of prioritized genes was identified in the AVM3 sample. Gene Ontology (GO) annotations for biological processes revealed that gene loci such as CTNNB1, COL3A1, NRP2, SLIT2, SLIT3, and SMO are implicated in vessel and heart morphogenesis. Specifically, the loci for the CTNNB1 and COL3A1 genes are involved in the TGF-β signaling pathways.

The SLIT2 gene was also identified in our study, consistent with the findings of Concetta Scimone et al. [14]. SLIT2 has been suggested to inhibit endothelial cell proliferation and migration during vascular development [32]. Furthermore, Hauer et al. performed RNA sequencing on bAVM patient samples and identified enriched GO terms related to cytoskeletal networks, cell migration, the endoplasmic reticulum, transmembrane transport, and extracellular matrix composition [33]. Gene loci such as MAPK3 are also involved in BMP signaling pathways.

The identified loci, including PARD3, OBSL1, ERBB2, and LRRK2 [34], are involved in endothelial/mesenchymal differentiation processes. GO enrichment analysis further grouped mutated genes according to the biological pathways they influence. Among these loci, the OBSL1 gene [35] encodes a cytoskeletal adaptor protein belonging to the Unc-89/obscurin family. Cytoskeletal adaptor proteins primarily function to link the internal cytoskeleton to the cellular membrane.

In Table 5, gene sets involved in biological processes, the disruption of which may contribute to the development of AVM, are presented. The CTNNB1 gene encodes β-catenin, a key protein involved in various cellular processes, including cell adhesion, gene transcription regulation, and signaling pathways. Its role in blood vessel morphogenesis is particularly significant due to its involvement in the Wnt signaling pathway, which is crucial for vascular development and remodeling. The CTNNB1 gene and its product, β-catenin, play a crucial role in blood vessel morphogenesis through their involvement in endothelial cell behavior, gene expression regulation, and interactions with the extracellular matrix [36] It should be noted that the genes COL3A1, CTNNB1, and ERBB2 are involved at all stages of vascular system development—angiogenesis, vasculogenesis, vascular morphogenesis, and regulation. COL3A1 and ENG are directly involved in maintaining vascular integrity, and mutations or dysregulation can lead to weakened vessel walls and increased susceptibility to malformations like AVMs. β-catenin can regulate the expression of genes involved in ECM remodeling, including those encoding collagens like COL3A1. This suggests a feedback mechanism in which β-catenin may influence the availability of type III collagen during angiogenesis [37].

Also known as HER2, ERBB2 (Erb-b2 receptor tyrosine kinase 2) is involved in cell signaling pathways that regulate cell growth and differentiation, including in endothelial cells during vessel formation. If a mutation in the ERBB2 gene is classified as “damaging” by the SIFT algorithm, it suggests that the mutation is likely to adversely affect the protein’s function. The mechanisms driving the development of AVMs involve abnormal angiogenesis (blood vessel formation) and remodeling. Since ERBB2 is involved in signaling pathways that regulate cell growth and differentiation, it could theoretically play a role in the pathophysiology of vascular malformations [38].

Pathogenic genetic variants do indeed play a key role in the pathogenesis of various diseases, including arteriovenous malformations (AVMs) of the brain. These variants can affect the development and functioning of the vascular system, which can in turn lead to the development of AVMs. Limited data are available regarding rs142456637, a polymorphism of the ERBB2 gene (Erb-b2 receptor tyrosine kinase 2). Most studies on ERBB2 polymorphisms focus on their association with cancer. More studies are needed to explore any potential associations between ERBB2 expression and the development or characteristics of brain AVMs. Moreover, the aforementioned genes are involved in the TGF-β signaling pathway. MAPK signaling pathways, including MAPK3, are involved in regulating angiogenesis (the formation of new blood vessels) and vascular remodeling [39]. Abnormalities in these processes can contribute to the development of vascular malformations, such as AVMs. Variants like rs55859133 may influence the expression or function of MAPK3, potentially affecting pathways related to angiogenesis and vascular health (there is a mutational spectrum of syndromic genes involved in sporadic brain arteriovenous malformation). SLIT3 is a gene that encodes a protein involved in various developmental processes, including vascular development. Research has indicated that SLIT3 mutations or dysregulation may be associated with arteriovenous malformations (AVMs), particularly in the brain. SLIT3 may influence angiogenesis (the formation of new blood vessels) and vascular stability. Abnormalities in these processes can contribute to the development of AVMs, which are characterized by an abnormal connection between arteries and veins, bypassing the capillary system [40]. The SMO gene (Smoothened) encodes a protein that is a key component of the Hedgehog signaling pathway. The SMO protein acts as a receptor for Hedgehog ligands, which are signaling molecules that initiate the Hedgehog signaling cascade [41]. This pathway is involved in regulating cell growth, differentiation, and tissue patterning during embryonic development. While the direct role of SMO in vascular malformations like arteriovenous malformations (AVMs) is not fully understood, the Hedgehog pathway has been shown to influence vascular development and remodeling. Dysregulation of this pathway could potentially contribute to abnormal blood vessel formation.

The TTN gene, also known as Titin, encodes a large protein that plays a crucial role in the structure and function of muscle tissues; it is involved in the positive regulation of sprouting angiogenesis, the regulation of cell adhesion, and the regulation of cell migration. Some investigations have been conducted into the genetic basis of brain AVMs, and although TTN has not been a primary focus, it could be included in broader studies examining the role of large genes in vascular development and integrity.

We discussed the loci affected by germline variants in five bAVM samples.

**Figure 3:**
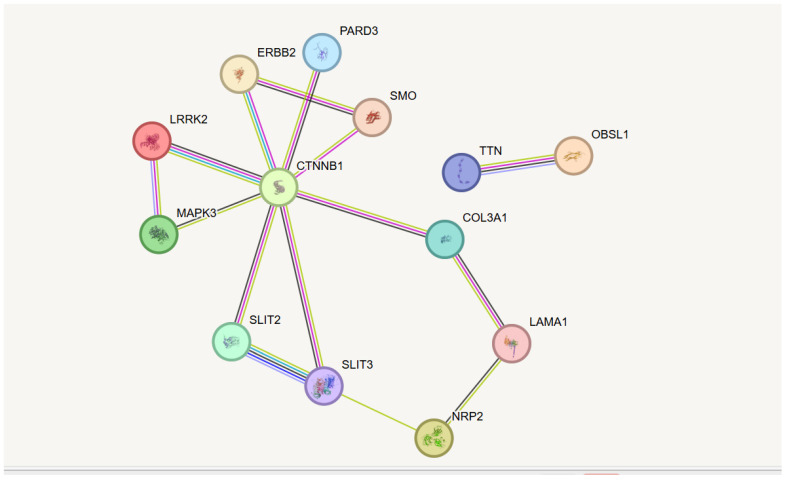
Functional network of prioritized genes. The image describes observed and inferred functional interactions linking prioritized genes. Nodes represent input proteins, while edges represent protein–protein associations. Note: known interaction (from curated databases)—light blue; gene co-occurrence—blue; experimentally determined—violet; textmining—light green; co-expression—black. The interaction network was constructed using the STRING tool, Version 12.0 (https://string-db.org/, accessed on 5 February 2025).

Although each examined sample presented distinct genes and genetic variants, all genes identified collectively participate in common biological processes and pathways (Table 6). Figure 3 illustrates the interactions among proteins encoded by these prioritized genes. From Figure 3, it is evident that the protein encoded by the CTNB1 gene interacts with almost all the proteins described in Table 6. The proteins encoded by TNN and OBSL1 fall out of the overall interaction cluster. The protein–protein interaction network, visualized using STRING, also shows involvement in biological pathways, primarily those related to cellular component morphogenesis. Detailed information about nodes and edges is provided in the Supplementary Materials.

The limitation of this study relates to the small number of samples examined; undoubtedly, the results require further validation in a larger cohort of patients.

## 5. Conclusions

The results of this study indicate that sporadic arteriovenous malformations (AVMs) in the brain are a heterogeneous condition. Thus, we selected only the loci affected by rare variants (MAF < 0.01) and then those that are most likely associated with the onset of the disease (brain AVM). Most candidate genes identified are involved in biological processes such as vessel development, TGF-β receptor signaling, BMP signaling, and endothelial/mesenchymal differentiation. Disruptions in these pathways may provide potential mechanisms underlying bAVM pathogenesis. In silico analysis further revealed candidate genes likely associated with lesion development, including COL3A1, CTNNB1, LAMA1, NPHP3, SLIT2, SLIT3, SMO, MAPK3, LRRK2, TTN, ERBB2, PARD3, and OBSL1. It is essential to focus on the genetic variants affecting the prioritized genes, ERBB2, SLIT3, SMO, MAPK3, and TTN, as mutations in these genes were predicted to be “damaging”. Most of these genes are involved in signaling pathways that control vasculogenesis and angiogenesis and work together through various signaling pathways to regulate the formation and remodeling of blood vessels during embryonic development and in response to physiological needs. Mutations or dysregulation of these genes can lead to vascular malformations, diseases, or conditions related to impaired vasculogenesis or angiogenesis. Understanding their roles can provide insights into potential therapeutic targets for vascular diseases.

However, further research is required to establish definitive correlations between genetic mutations and clinical phenotypes.

## Figures and Tables

**Table 1:** Anamnestic data for patients with bAVM.

Patient ID	Gender	Age (Years)	Spetzler–Martin Grade	Presenting Symptoms	Lesion Location	Family History of bAVM	Previous Hemorrhage
AVM1	Female	37	II	Cephalalgia, generalized weakness, and seizures	Left frontal lobe	no	no
AVM2	Male	40	III	Weakness in the left extremities and seizures	Right parietal lobe	no	no
AVM3	Female	25	II	Vertigo and tinnitus	Right temporal lobe	no	no

**Table 2:** Fastq statistics.

Sample ID	Total Yield (bp)	Total Reads	GC (%)	AT (%)	Q20 (%)	Q30 (%)
AVM1	7,984,584.64	452,878,044	52.04	47.96	97.33	93.05
AVM2	7,478,531.09	649,526,696	51.88	48.12	97.2	92.76
AVM3	6,884,648.39	845,593,698	51.56	48.44	97.21	92.82

Sample ID—sample name; Total Yield (bp)—total number of bases sequenced; Total Reads—total number of reads; GC (%)—GC content; AT (%)—AT content; Q20 (%)—ratio of bases that have a Phred quality score of over 20; Q30 (%)—ratio of bases that have a Phred quality score of over 30.

**Table 3:** Number of reads, coverage, and variant statistics by sample.

Sanple ID	AVM1	AVM2	AVM3
Total reads	52,876,354	49,524,628	45,592,274
Average read length (bp)	149.38	149.54	148.93
Number of on-target genotypes (≥1×)	60,319,451	60,315,825	60,455,735
% Coverage of target regions (≥10×)	99.6	99.6	99.9
% Coverage of target regions (≥30×)	82.3	80.4	77.6
% Coverage of target regions (≥50×)	57.0	53.2	58.7
Number of SNPs	100,575	99,054	99,231
Missense Variants	12,225	12,146	12,280
Stop Gained	135	120	120
Stop Lost	25	27	33
Number of INDELs	14,140	15,754	15,628
Frameshift Variants	275	291	282
% Found in dbSNP151	98.9	99.0	99.0

**Table 4:** ClueGO (Gene Ontology) enrichment analysis results.

Sample ID	GO ID	Term BP	*p*-Value with Bonferroni Correction	Genes
**AVM1**				
	GO:0035469	determination of pancreatic left/right asymmetry	0.035	CCDC40; DNAAF1; NPHP3; DNAAF2; ZMYND10
	GO:0036159	inner dynein arm assembly	0.035	CCDC40; DNAAF1; NPHP3
	GO:0071910	determination of liver left/right liver asymmetry	0.035	CCDC40; DNAAF1; NPHP3
	GO:0071907	determination of digestive tract left/right asymmetry	0.035	CCDC40; DNAAF1; DNAAF2; DNAH8; ZMYND10
	GO:0070286	axonemal dynein complex assembly		CCDC40; DNAAF1; DNAAF2; DNAH8; ZMYND10
	GO:0036158	outer dynein arm assembly	0.035	DNAAF1; DNAAF2; DNAH8; ZMYND10
	GO:2001044	regulation of integrin-mediated signaling pathway	0.035	BST1; CD177; LAMA1; LMNB2
	GO:0003351	epithelial cilium movement involved in extracellular fluid	0.035	BST1; CD177; LAMA1; LMNB2 CCDC40; DNAAF1; DNAAF2; NPHP3; STK36
	GO:0006858	extracellular transport	0.035	CCDC40; DNAAF1; DNAAF2; NPHP3; STK36
**AVM2**				
	GO:0006536	glutamate metabolic process	0.0426	FTCD; PRODH
	GO:0045222	CD4 biosynthetic process	0.0036	ACOT8
	GO:0045223	regulation of CD4 production	0.0036	ACOT8
	GO:0045225	negative regulation of CD4 production	0.0036	ACOT8
**AVM3**				
	GO:0007156	homophilic cell adhesion via plasma membrane adhesion molecules	0.00049	STK36
	GO:0048667	cell morphogenesis involved in neuron differentiation	0.00002	CCDC40; DNAAF1; DNAAF2; NPHP3;
	GO:0055003	cardiac myofibril assembly	0.00026	STK36
	GO:0048790	maintenance of presynaptic active zone structure	0.00030	BSN; CTBP2; PCLO
	GO:0098882	structural constituent of presynaptic active zone	0.00030	BSN; CTBP2; PCLO
	GO:1904415	regulation of xenophagy	0.00049	LRSAM1; MAPK3; RNF31
	GO:1904417	positive regulation of xenophagy	0.00049	LRSAM1; MAPK3; RNF31
	GO:0021885	forebrain cell migration	0.00029	COL3A1; CTNNB1; DISC1; LRRK2; RTN4; SLIT2; SLIT3
	GO:0022029	telencephalon cell migration	0.00029	COL3A1; CTNNB1; DISC1; LRRK2; RTN4; SLIT2; SLIT3
	GO:0022028	tangential migration from subventricular zone to olfactory bulb	0.00029	LRRK2; SLIT2; SLIT3

Note: This table reports annotations from the ClueGO enrichment analysis. For each sample, the enriched pathways (GO Term; third column) and clustered genes (fifth column) are provided.

**Table 5:** Genes prioritized using ToppGene tool.

Ontology	Feature	ID	Name	Genes
GO: Biological Process	**Vessel development**			
		GO:0001569	branching involved in blood vessel morphogenesis	**CTNNB1** ENG GDF2 TGFBR2
		GO:0048514	blood vessel morphogenesis	ACVRL1 **COL3A1 CTNNB1** ENG ERBB2 GDF2 **LAMA1 NRP2 SLIT2 SMO** TGFBR2
		GO:0001568	blood vessel development	ACVRL1 **COL3A1 CTNNB1** ENG ERBB2 GDF2 **LAMA1 NRP2 SLIT2 SMO** TGFBR2
		GO:0001570	vasculogenesis	**CTNNB1** ENG GDF2 **SMO** TGFBR2
		GO:0001525	angiogenesis	ACVRL1 **CTNNB**1 ENG **ERBB2** GDF2 **NRP2 SLIT2** TGFBR2
		GO:0045765	regulation of angiogenesis	ACVRL1 **CTNNB1** ENG **ERBB2** GDF2 TGFBR2
		GO:0001944	vasculature development	ACVRL1 **COL3A1 CTNNB1** ENG **ERBB2** GDF2 **LAMA1 NRP2 SLIT2 SMO** TGFBR2
		GO:0035295	tube development	ACVRL1 **COL3A1 CTNNB1** ENG **ERBB2** GDF2 **LAMA1 MAPK3 NRP2 SLIT2** SMAD4 **SMO** TGFBR2
		GO:0035239	tube morphogenesis	ACVRL1 **COL3A1 CTNNB1** ENG **ERBB2** GDF2 **LAMA1 NRP2 SLIT2** SMAD4 **SMO** TGFBR2
		GO:1901342	regulation of vasculature development	ACVRL1 **CTNNB1** ENG **ERBB2** GDF2 TGFBR2
		GO:0035909; GO:0035904	aorta development	ACVRL1 **COL3A1** ENG TGFBR2
		GO:0048844	artery morphogenesis	ACVRL1 **COL3A1 CTNNB1** ENG TGFBR2
	** Heart development **			
		GO:0003007	heart morphogenesis	ACVRL1 **CTNNB1** ENG **NRP2 SLIT2 SLIT3** SMAD4 **SMO** TGFBR2 **TTN**
		GO:2000136	regulation of cell proliferation involved in heart morphogenesis	**CTNNB1** ENG SMAD4
		GO:0061323	cell proliferation involved in heart morphogenesis	**CTNNB1** ENG SMAD4 TGFBR2
		GO:0003148	outflow tract septum morphogenesis	ENG **NRP2** SMAD4 TGFBR2
		GO:0003181; GO:0003171	atrioventricular valve morphogenesis	**SLIT3** SMAD4 TGFBR2
		GO:0003208	cardiac ventricle morphogenesis	**CTNNB1** ENG SMAD4 TGFBR2
		GO:0060411	cardiac septum morphogenesis	ENG **NRP2 SLIT2 SLIT3** SMAD4 **SMO** TGFBR2
		GO:0003279	cardiac septum development	ENG **NRP2 SLIT2 SLIT3** SMAD4 **SMO** TGFBR2
	**BMP signaling**			
		GO:0030509	BMP signaling pathway	ACVRL1 ENG GDF2 **MAPK3** SMAD4
		GO:0071772	response to BMP	ACVRL1 ENG GDF2 **MAPK3** SMAD4
		GO:0071773	cellular response to BMP stimulus	ACVRL1 ENG GDF2 **MAPK3** SMAD4
	** TGFBR signaling **			
		GO:0007179	transforming growth factor beta receptor signaling pathway	ACVRL1 **COL3A1** ENG GDF2 SMAD4 TGFBR2
		GO:0071560	cellular response to transforming growth factor beta stimulus	ACVRL1 **COL3A1** ENG GDF2 SMAD4 TGFBR2
		GO:0071559	response to transforming growth factor beta	ACVRL1 **COL3A1** ENG GDF2 SMAD4 TGFBR2
		GO:0090287	regulation of cellular response to growth factor stimulus	ACVRL1 **CTNNB1** ENG GDF2 **SLIT2** SMAD4
		GO:0009719	response to endogenous stimulus	ACVRL1 **COL3A1 CTBP2 CTNNB1** ENG **ERBB2** GDF2 **LRRK2 MAPK3 NRP2 SLIT2** SMAD4 TGFBR2
		GO:0141091	transforming growth factor beta receptor superfamily signaling pathway	ACVRL1 **COL3A1** ENG GDF2 **MAPK3** SMAD4 TGFBR2
	** Endothelial/mesenchymal differentiation **			
		GO:0060429	epithelium development	ACVRL1 **CDH23 CTNNB1** ENG GDF2 **LAMA1 PARD3 SLIT2** SMAD4 **SMO** TGFBR2
		GO:0048754	branching morphogenesis of an epithelial tube	**CTNNB1** ENG GDF2 **LAMA1 SLIT2** SMAD4 **SMO** TGFBR2
		GO:0045603	positive regulation of endothelial cell differentiation	ACVRL1 **CTNNB1** GDF2
		GO:0010718	positive regulation of epithelial to mesenchymal transition	**CTNNB1** ENG SMAD4 TGFBR2
		GO:0010717	regulation of epithelial to mesenchymal transition	**CTNNB1** ENG SMAD4 TGFBR2
		GO:0045595	regulation of cell differentiation	ACVRL1 **CTNNB1** ENG **ERBB2** GDF2 **LAMA1 LRRK2 OBSL1 SLIT2** SMAD4 **SMO** TGFBR2
		GO:0045446	endothelial cell differentiation	ACVRL1 **CTNNB1** GDF2 SMAD4
		GO:0003158	endothelium development	ACVRL1 **CTNNB1** GDF2 SMAD4
		GO:0001837	epithelial to mesenchymal transition	ACVRL1 **CTNNB1** ENG SMAD4 TGFBR2
		GO:0001936	regulation of endothelial cell proliferation	ACVRL1 ENG GDF2 **NRP2**
		GO:0050680	negative regulation of epithelial cell proliferation	ACVRL1 ENG GDF2 **SMO**
		GO:0048762	mesenchymal cell differentiation	ACVRL1 **CTNNB1** ENG **MAPK3 NRP2** SMAD4 **SMO** TGFBR2
		GO:0048863	stem cell differentiation	**CTNNB1** ENG **MAPK3 NRP2** SMAD4 **SMO** TGFBR2
		GO:0060485	mesenchyme development	ACVRL1 **CTNNB1** ENG **MAPK3 NRP2** SMAD4 **SMO** TGFBR2
		GO:0060562	epithelial tube morphogenesis	ACVRL1 **CTNNB1** ENG GDF2 **LAMA1 SLIT2** SMAD4 **SMO** TGFBR2
		GO:0010631	epithelial cell migration	ACVRL1 GDF2 **NRP2 SLIT2** SMAD4 TGFBR2
		GO:0090132	epithelium migration	ACVRL1 GDF2 **NRP2 SLIT2** SMAD4 TGFBR2
		GO:0045595	regulation of cell differentiation	ACVRL1 **CTNNB1** ENG **ERBB2** GDF2 **LAMA1 LRRK2 OBSL1 SLIT2** SMAD4 **SMO** TGFBR2
		GO:0045595	regulation of cell differentiation	ACVRL1 **CTNNB1** ENG **ERBB2** GDF2 **LAMA1 LRRK2 OBSL1 SLIT2** SMAD4 **SMO** TGFBR2
		GO:0090130	tissue migration	ACVRL1 GDF2 **NRP2 SLIT2** SMAD4 TGFBR2
		GO:0002009	morphogenesis of an epithelium	ACVRL1 **CTNNB1** ENG GDF2 **LAMA1 PARD3 SLIT2** SMAD4 **SMO** TGFBR2

Note: This table presents the results of the prioritization analysis conducted using ToppGene, utilizing the genetic set of genes ACVRL1, ENG, GDF2, SMAD4, and TGFBR2 as the training set. The prioritized genes are highlighted in bold according to the specific ontology. The results are summarized and grouped by annotations.

**Table 6:** Characterization of genetic variants affecting prioritized genes.

№	Gene_Name	Chromosome	HGVS.c	HGVS.p	dbSNP151_ID	p3_1000G_AF	SIFT_pred
1	CTNNB1	chr3	c.2129G>A	p.Arg710His	rs200308943	0.0001997	T
2	COL3A1	chr2	c.3133G>A	p.Ala1045Thr	rs149722210	0.0069	T
3	ERBB2	chr17	c.1466C>T	p.Pro489Leu	rs142456637	0.000399	**D**
4	LAMA1	chr18	c.181G>A	p.Val61Ile	rs147676957	0.0002	T
5	NRP2	chr2	c.962A>T	p.Asn321Ile	rs151124318	0.001198	T;D;
6	SLIT3	chr5	c.1184G>A	p.Arg395Gln	rs2288792	0.0045927	D
7	SLIT2	chr4	c.4253C>T	p.Ala1418Val	rs143417693	0.0003994	T
8	SMO	chr7	c.536C>T	p.Thr179Met	rs115491500	0.001398	**D**
9	PARD3	chr10	c.2402G>A	p.Ser801Asn	rs118153230	0.0085863	T
10	LRRK2	chr12	c.7153G>A	p.Gly2385Arg	rs34778348	0.004792	T
11	OBSL1	chr2	c.4361G>A	p.Arg1454Gln	rs183329050	0.004792	T
12	MAPK3	chr16	c.967G>A	p.Glu323Lys	rs55859133	0.000599042	D
13	TTN	chr2	c.14902G>A	p.Asp4968Asn	rs371444691	0.000199681	D

Note: SIFT was used to predict the pathogenicity of missense mutations. The SIFT (Sorting Intolerant From Tolerant) algorithm is a widely used computational tool that predicts the potential impact of amino acid substitutions on protein function based on the evolutionary conservation of the affected amino acid. If the SIFT score is smaller than 0.05, the corresponding nsSNV is predicted as D (damaging); otherwise, it is predicted as T (tolerated).

**Figure A2:**
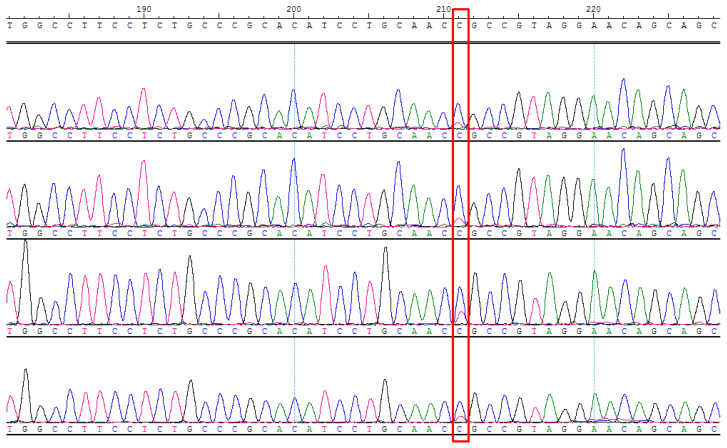
Validation analysis of the candidate variants (p.Arg1454Gln, rs183329050) in the genes OBSL1.

**Figure A3:**
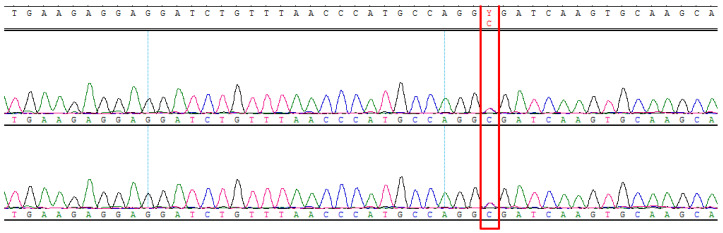
Validation analysis of the candidate variants (p.Ala1418Val, rs143417693) in the genes SLIT2.

**Figure A4:**
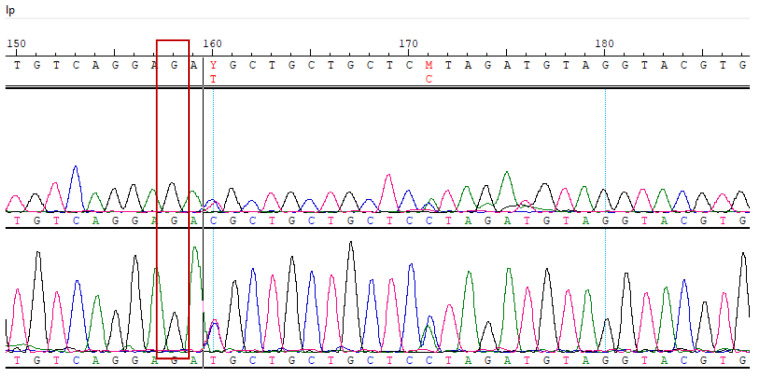
Validation analysis of the candidate variants (p.Pro489Leu, rs142456637) in the genes ERBB2.

**Figure A5:**
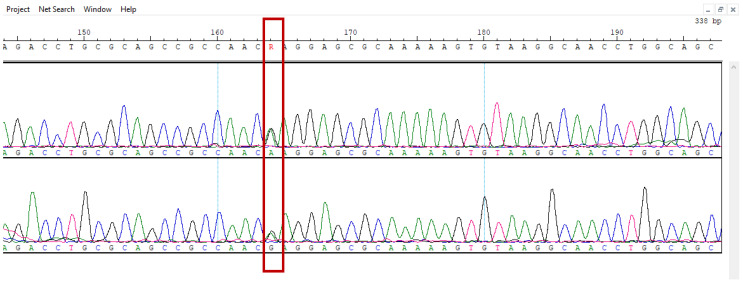
Validation analysis of the candidate variants (p.Arg710His, rs200308943) in the genes CTNNB1.

**Figure A6:**
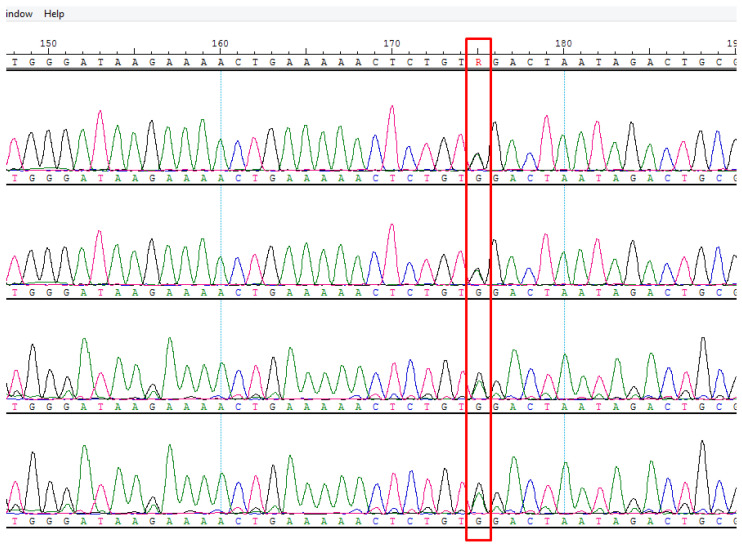
Validation analysis of the candidate variants (p.Ala1045Thr, rs149722210) in the genes COL3A1.

**Figure A7:**
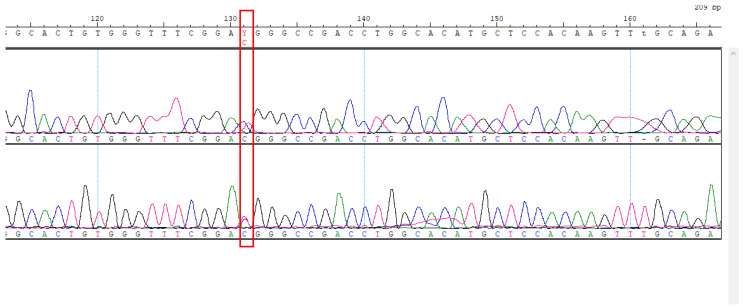
Validation analysis of the candidate variants (p.Val61Ile, rs147676957) in the genes LAMA1.

**Figure A8:**
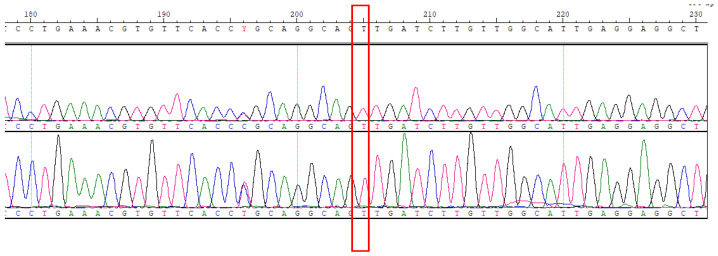
Validation analysis of the candidate variants (p.Arg395Gln, rs2288792) in the genes SLIT3.

**Figure A9:**
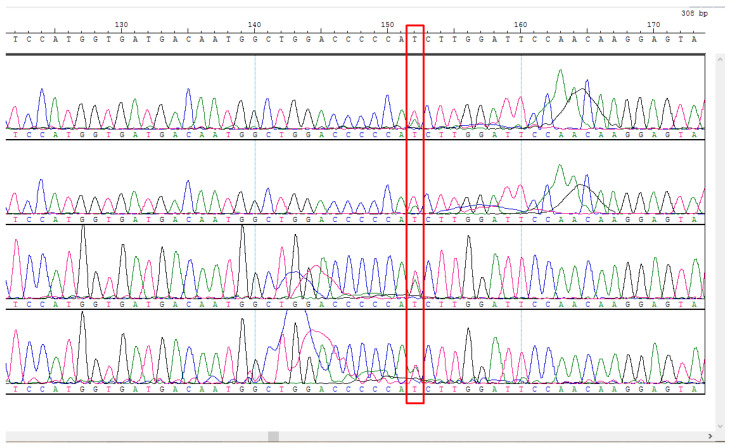
Validation analysis of the candidate variants (p.Asn321Ile, rs151124318) in the genes NRP2.

**Figure A10:**
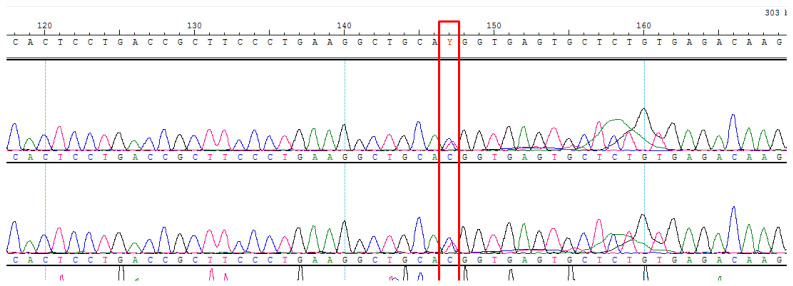
Validation analysis of the candidate variants p.Thr179Met, rs115491500) in the genes SMO.

**Figure A11:**
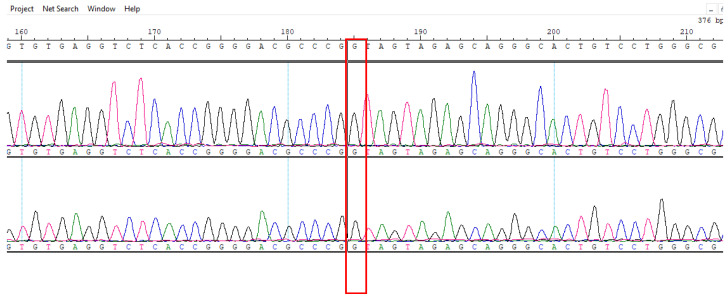
Validation analysis of the candidate variants p.Arg98Trp, rs143386487) in the genes. LAMA5 (control (conditionally healthy).

## Data Availability

Data can be provided upon request.

## Supplementary Materials

The following supporting information can be downloaded at: https://www.mdpi.com/article/10.3390/biomedicines13061451/s1, Table S1: AVM1; Table S2: AVM2; Table S3: AVM3.

## Appendix A

### Appendix A.1. Primer Sequences and PCR Conditions

**Table A1:** Primer sequences used for Sanger sequencing.

Name Primers	Sequence (5″–3″)	Tm (°C)	Amplicon Length (bp)
rs149722210-F	CCTGGATCAGATGGTCTTCC	59.1	395
rs149722210-R	CACACATTTGTCTAAGGAACAACTA	59	
rs143417693-F	TTCTCAATCATCCTCCATTCTT	54.6	425
rs143417693-R	CTCTCTGTCTCTGTTGCTC	58.1	
rs34778348-F	TTAAGAAGAAAACAAATAGTGATGAC	56	361
rs34778348-R	TGATCTGAAAAGATGGTGCT	56.6	
rs200308943-F	AGGTGTGGAGTTTTGAAGAA	58.4	438
rs200308943-R	GTGGTGTAATCCTACTGCT	57.6	
rs142456637-F	GGATGGAGGAAGATGAGAATAG	58.7	461
rs142456637-R	AAGGCAGGTAGGACCCAG	61.9	
rs115491500-F	ATGCCCAAGTGTGAGAATGA	60.9	301
rs115491500-R	GCAGTTTGAGTTTGTGTCCT	60.1	
rs118153230-F	AGTGAATATGCCCCAAGAT	57.3	286
rs118153230-R	AAAACCATGAAGACAGAGC	56.9	
rs151124318-F	CTTTCCCCAGACTTTCAGT	58.1	307
rs151124318-R	TGAATGGAGTTCTCAGGTATG	58.3	
rs2288792-F	TAGACACAGGAAGGCAGGT	61.4	393
rs2288792-R	CCTCATTTGGGTGTTTTTCATG	59.9	
rs183329050-F	GATGAGGATGAGATACTCTGTGTC	61.3	386
rs183329050-R	CTCTCGCAGCCGTAGGTG	63.7	
NM_005559.3_F	ATCAGCACCAATGCCACCT	63.5	206
NM_005559.3_R	CCTAACAGAAGTCTCAGTCCTC	60.8	
rs55859133-F	CCAAGTCAGACTCCAAAGGT	59	446
rs55859133-R	TTCAGCCGCTCCTTAGGTA	59.8	
rs371444691-F	ACTGAGGCAGCAGAATCTGA	62.5	426
rs371444691-R	TTCAGCAACTTCCCCTAAAGG	61.5	

PCR conditions: The PCR reaction was performed with primers from Table A1 s149722210F-cctggatcagatggtcttcc in a total volume of 20 µL. The PCR mixture contained 50 ng. DNA, 0.2 Units. TaqPolymerase (Syntol, Novosibirsk, Russia), 2 mM each dNTP, 10× PCR buffer (Thermo Fisher), 25 mM MgCl_2_ (Thermo Fisher), 10 pmol each primer. The PCR amplification program included long-term denaturation at 95 °C for 5 min; 30 cycles: 95 °C—30 s, annealing temperature from Table A1—40 s, 72 °C—1 min; final elongation 10 min at 72 °C; PCR program was performed using a VeritiPro by Thermo Fisher Scientific.

### Appendix A.3

**Table A2:** Statistically significant genes prioritized by ToppGene tool.

Gene Symbol	Gene ID	GO: Biological Process		Human Phenotype		Mouse Phenotype		Pathway		Interaction		Disease		Average Score	Overall *p*-Value
		Score	*p* Value	Score	*p* Value	Score	*p* Value	Score	*p* Value	Score	*p* Value	Score	*p* Value		
**AVM1**															
DNAAF2	55172	6.083 × 10^−1^	2.027 × 10^−2^	7.941 × 10^−1^	5.232 × 10^−3^	9.114 × 10^−1^	1.000 × 10^−6^	0.000 × 10^0^	5.043 × 10^−1^	0.000 × 10^0^	5.062 × 10^−1^	0.000 × 10^0^	5.010 × 10^−1^	3.856 × 10^−1^	3.381 × 10^−4^
LAMA1	284217	9.995 × 10^−1^	5.886 × 10^−3^	0.000 × 10^0^	5.049 × 10^−1^	6.482 × 10^−1^	6.540 × 10^−4^	0.000 × 10^0^	5.043 × 10^−1^	0.000 × 10^0^	5.062 × 10^−1^	0.000 × 10^0^	5.010 × 10^−1^	2.746 × 10^−1^	1.870 × 10^−2^
NPHP3	27031	9.984 × 10^−1^	7.848 × 10^−3^	9.588 × 10^−1^	2.616 × 10^−3^	0.000 × 10^0^	5.056 × 10^−1^	0.000 × 10^0^	5.043 × 10^−1^	0.000 × 10^0^	5.062 × 10^−1^	0.000 × 10^0^	5.010 × 10^−1^	3.262 × 10^−1^	3.532 × 10^−2^
DNAAF1	123872	9.890 × 10^−1^	8.502 × 10^−3^	7.941 × 10^−1^	5.232 × 10^−3^	0.000 × 10^0^	5.056 × 10^−1^	0.000 × 10^0^	5.043 × 10^−1^	0.000 × 10^0^	5.062 × 10^−1^	0.000 × 10^0^	5.010 × 10^−1^	2.972 × 10^−1^	4.702 × 10^−2^
CCDC40	55036	9.890 × 10^−1^	8.502 × 10^−3^	7.941 × 10^−1^	5.232 × 10^−3^	0.000 × 10^0^	5.056 × 10^−1^	0.000 × 10^0^	5.043 × 10^−1^	0.000 × 10^0^	5.062 × 10^−1^	0.000 × 10^0^	5.010 × 10^−1^	2.972 × 10^−1^	4.702 × 10^−2^
**AVM2**															
PRODH	5625	0.000 × 10^0^	5.036 × 10^−1^	0.000 × 10^0^	5.199 × 10^−1^	0.000 × 10^0^	5.016 × 10^−1^	0.000 × 10^0^	5.062 × 10^−1^	0.000 × 10^0^	5.056 × 10^−1^	0.000 × 10^0^	5.029 × 10^−1^	2.999 × 10^−2^	2.793 × 10^−1^
**AVM3**															
CTNNB1	1499	1.000 × 10^0^	1.308 × 10^−3^	1.000 × 10^0^	1.000 × 10^−6^	6.482 × 10^−1^	1.308 × 10^−3^	9.999 × 10^−1^	1.000 × 10^−6^	9.538 × 10^−1^	1.000 × 10^−6^	8.861 × 10^−1^	6.540 × 10^−4^	7.840 × 10^−1^	4.256 × 10^−11^
COL3A1	1281	1.000 × 10^0^	1.308 × 10^−3^	1.000 × 10^0^	1.000 × 10^−6^	4.011 × 10^−1^	4.578 × 10^−3^	0.000 × 10^0^	5.023 × 10^−1^	8.651 × 10^−1^	6.540 × 10^−4^	3.839 × 10^−1^	1.243 × 10^−2^	6.036 × 10^−1^	1.437 × 10^−7^
ERBB2	2064	9.998 × 10^−1^	1.308 × 10^−3^	7.898 × 10^−1^	7.194 × 10^−3^	8.202 × 10^−1^	1.308 × 10^−3^	9.337 × 10^−1^	6.540 × 10^−4^	2.399 × 10^−1^	7.194 × 10^−3^	9.559 × 10^−1^	6.540 × 10^−4^	6.958 × 10^−1^	2.431 × 10^−6^
SMO	6608	1.000 × 10^0^	1.308 × 10^−3^	9.934 × 10^−1^	1.962 × 10^−3^	8.858 × 10^−1^	6.540 × 10^−4^	0.000 × 10^0^	5.023 × 10^−1^	7.430 × 10^−1^	2.616 × 10^−3^	0.000 × 10^0^	5.108 × 10^−1^	5.886 × 10^−1^	1.922 × 10^−5^
MAPK3	5595	9.999 × 10^−1^	1.308 × 10^−3^			0.000 × 10^0^	5.029 × 10^−1^	1.000 × 10^0^	1.000 × 10^−6^	8.696 × 10^−1^	6.540 × 10^−4^	0.000 × 10^0^	5.108 × 10^−1^	4.783 × 10^−1^	4.221 × 10^−5^
TTN	7273	9.068 × 10^−1^	1.112 × 10^−2^	8.880 × 10^−1^	3.924 × 10^−3^	9.274 × 10^−1^	1.000 × 10^−6^	0.000 × 10^0^	5.023 × 10^−1^	2.399 × 10^−1^	7.194 × 10^−3^	0.000 × 10^0^	5.108 × 10^−1^	4.374 × 10^−1^	6.651 × 10^−5^
PARD3	56288	6.707 × 10^−1^	2.158 × 10^−2^			5.070 × 10^−1^	3.924 × 10^−3^	7.114 × 10^−1^	6.540 × 10^−4^	2.399 × 10^−1^	7.194 × 10^−3^	3.839 × 10^−1^	1.243 × 10^−2^	4.655 × 10^−1^	1.632 × 10^−4^
RNF31	55072	2.066 × 10^−1^	3.924 × 10^−2^	9.516 × 10^−2^	1.439 × 10^−2^	5.070 × 10^−1^	3.924 × 10^−3^	0.000 × 10^0^	5.023 × 10^−1^	6.430 × 10^−1^	3.924 × 10^−3^	8.499 × 10^−1^	1.308 × 10^−3^	3.947 × 10^−1^	3.966 × 10^−4^
CTBP2	1488	6.164 × 10^−1^	2.158 × 10^−2^			8.980 × 10^−1^	6.540 × 10^−4^	2.781 × 10^−1^	4.578 × 10^−3^	2.399 × 10^−1^	7.194 × 10^−3^	0.000 × 10^0^	5.108 × 10^−1^	4.084 × 10^−1^	8.475 × 10^−4^
SLIT2	9353	1.000 × 10^0^	1.308 × 10^−3^			5.726 × 10^−1^	3.270 × 10^−3^	0.000 × 10^0^	5.023 × 10^−1^	2.399 × 10^−1^	7.194 × 10^−3^	0.000 × 10^0^	5.108 × 10^−1^	3.021 × 10^−1^	3.614 × 10^−3^
CDH23	64072	1.974 × 10^−1^	3.924 × 10^−2^	9.929 × 10^−1^	1.962 × 10^−3^	0.000 × 10^0^	5.029 × 10^−1^	0.000 × 10^0^	5.023 × 10^−1^	2.399 × 10^−1^	7.194 × 10^−3^	3.497 × 10^−1^	1.831 × 10^−2^	2.900 × 10^−1^	4.633 × 10^−3^
NRP2	8828	1.000 × 10^0^	1.308 × 10^−3^			0.000 × 10^0^	5.029 × 10^−1^	0.000 × 10^0^	5.023 × 10^−1^	6.654 × 10^−1^	3.270 × 10^−3^	3.680 × 10^−1^	1.700 × 10^−2^	3.389 × 10^−1^	5.090 × 10^−3^
ARHGEF25	115557	0.000 × 10^0^	5.193 × 10^−1^			0.000 × 10^0^	5.029 × 10^−1^	0.000 × 10^0^	5.023 × 10^−1^	6.308 × 10^−1^	4.578 × 10^−3^	8.830 × 10^−1^	6.540 × 10^−4^	2.523 × 10^−1^	1.709 × 10^−2^
CELSR2	1952	4.949 × 10^−1^	2.485 × 10^−2^			0.000 × 10^0^	5.029 × 10^−1^	0.000 × 10^0^	5.023 × 10^−1^	2.399 × 10^−1^	7.194 × 10^−3^	4.493 × 10^−1^	9.810 × 10^−3^	2.321 × 10^−1^	1.790 × 10^−2^
LRRK2	120892	9.253 × 10^−1^	1.112 × 10^−2^	6.594 × 10^−2^	1.439 × 10^−2^	0.000 × 10^0^	5.029 × 10^−1^	0.000 × 10^0^	5.023 × 10^−1^	2.399 × 10^−1^	7.194 × 10^−3^	0.000 × 10^0^	5.108 × 10^−1^	2.084 × 10^−1^	2.159 × 10^−2^
SLIT3	6586	9.999 × 10^−1^	1.308 × 10^−3^			0.000 × 10^0^	5.029 × 10^−1^	0.000 × 10^0^	5.023 × 10^−1^	2.399 × 10^−1^	7.194 × 10^−3^	0.000 × 10^0^	5.108 × 10^−1^	2.066 × 10^−1^	2.633 × 10^−2^
OBSL1	23363	9.476 × 10^−1^	1.046 × 10^−2^	9.099 × 10^−2^	1.439 × 10^−2^	0.000 × 10^0^	5.029 × 10^−1^	0.000 × 10^0^	5.023 × 10^−1^	0.000 × 10^0^	5.062 × 10^−1^	3.497 × 10^−1^	1.831 × 10^−2^	2.321 × 10^−1^	2.957 × 10^−2^
